# Adaptive estimation of continuous gait phase based on capacitive sensors

**DOI:** 10.1017/wtc.2022.4

**Published:** 2022-06-17

**Authors:** Dongfang Xu, Zhitong Zhang, Simona Crea, Nicola Vitiello, Qining Wang

**Affiliations:** 1 Department of Advanced Manufacturing and Robotics, College of Engineering, Peking University, Beijing 100871, China; 2 Beijing Engineering Research Center of Intelligent Rehabilitation Engineering, Beijing, China; 3 National Key Laboratory of Science and Technology on Micro/Nano Fabrication, Institute of Micro/Nano Electronics, Peking University, Beijing, China; 4 BioRobotics Institute, Scuola Superiore Sant’Anna, Pisa, Italy; 5 Department of Excellence in Robotics & AI, Scuola Superiore Sant’Anna, Pisa, Italy; 6 IRCCS Fondazione Don Carlo Gnocchi, Milan, Italy; 7 Institute for Artificial Intelligence, Peking University, Beijing, China; 8 University of Health and Rehabilitation Sciences, Qingdao, China

**Keywords:** continuous gait phase, capacitive sensors, robotic transtibial prosthesis

## Abstract

Continuous gait phase plays an important role in robotic prosthesis control. In this paper, we have conducted the offline adaptive estimation (at different speeds and on different ramps) of continuous gait phase of robotic transtibial prosthesis based on the adaptive oscillators. We have used the capacitive sensing method to record the deformation of the muscles. Two transtibial amputees joined in this study. Based on the strain signals of the prosthetic foot and the capacitive signals of the residual limb, the maximum and minimum of estimation errors are 0.80 rad and 0.054 rad, respectively, and their corresponding ratios in one gait cycle are 1.27% and 0.86%, respectively. This paper proposes an effective method to estimate the continuous gait phase based on the capacitive signals of the residual muscles, which provides a basis for the continuous control of robotic transtibial prosthesis.

## Introduction

Lower-limb robotic prostheses can assist amputees’ daily activities. The control of robotic prosthesis is one critical issue, and the widely used control approach in lower-limb robotic prostheses is the finite state machine method (Sup et al., 2008; Au et al., 2009; Wang et al., 2015). This method is to build different control strategies corresponding to the several different gait phases within each gait cycle (Sup et al., 2009; Lenzi et al., 2014; Feng and Wang, 2017). Therefore, it relies on the accurate detection of gait events (heel strike, toe off, and so on) to trigger state transitions between different states, which could limit the smoothness and robustness of control (Villarreal et al., 2017; Yan et al., 2017).

To solve these problems, several alternatives have been proposed in recent years. Eilenberg et al. (2010) have designed the neuromuscular controller. The neuromuscular controller is to use models of muscle dynamics and hypothesized reflexes. However, this method involves many parameters which are difficult to tune. Another alternative is based on the continuous gait phase. Quintero et al. (2018) have conducted the continuous-phase control of a powered knee-ankle prosthesis and achieved some effects. Seo et al. (2019) have also conducted continuous gait phase estimation by recurrent neural network (RNN) method to control ankle exoskeletons. These control methods are based on the estimated continuous gait phase.

Continuous gait phase increases monotonically from 0 to 2 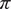 rad (or 0 to 100%) in each gait cycle. For the moment, there are three methods in the continuous gait phase estimation. The first method is to calculate the average duration of several previous gait cycles as the denominator, and then calculating the time percent (i.e., the gait phase, from 0 to 100%) relative to the average duration in each gait cycle. This method is easy and simple, however, the estimation accuracy based on this method may encounter decline for the lower-limb amputees, since there may be stride-to-stride variation for lower-limb amputees, even in the steady walking mode, which can cause the difference between the average duration and the current gait cycle time length. The second method is designing or utilizing a specific algorithm to estimate continuous gait phase, such as the adaptive oscillators (AOs) (Yan et al., 2017; Xu et al., 2020b) and the extended Kalman filter (Thatte et al., 2019). Xu et al., 2020b) have used the inertial measurement unit (IMU) signals to estimate the continuous gait phase of robotic transtibial prosthesis, and their study has shown some estimation adaptation to different walking conditions based on the AOs. Thatte et al. (2019) have used the extended Kalman filter to estimate the continuous gait phase in the stance phase (namely starting at heel strike (0%) and ending at toe off (100%)) based on IMU and angle signals of powered transfemoral prosthesis. The third method is based on the polar angle method (Holgate et al., 2009). Holgate et al. (2009) have found that the polar angle between the tibia angle and its scaled angular velocity has an invertible relationship with the gait phase and is not subject-dependent, and they have built a fitting function between the polar angle and gait phase and realized the continuous gait phase estimation. Quintero et al. (2018) have computed the continuous phase utilizing the thigh angular position and its corresponding integral to form a well-defined thigh orbit. The third method has good adaptations because of its subject-independent features, whereas its performance is susceptible to signal drift and integral drift and it is also sensitive to stride-to-stride gait variation. It is important to solve the gait variation problem. As known, the stride-to-stride gait variation can cause sensing signal variation. The property of AOs refers to the capacity to synchronize to an input sensing signal while learning its features (frequency, amplitude, etc.; Buchli et al., 2008; Ronsse et al., 2013). The error between the estimated signal (output) and the actual sensing signal (input) drives the evolution of the dynamic system to keep its output in phase with respect to the input signal (Ronsse et al., 2010; Yan et al., 2017). Therefore, the AOs can adjust the estimated gait phase when facing the stride-to-stride gait variation and handle this variation better in continuous gait phase estimation.

In addition to the estimation method, it is also very important to choose the proper sensing signals. The mechanical or inertial sensors have been used in gait phase estimation, for example, the IMU (Villarreal et al., 2017). However, these sensors may easily encounter wearing gap or misalignment problems. Surface electromyogram (sEMG) sensors can record muscles’ electrical potential and are widely used in robotic prosthesis. However, sEMG signals are easy to be contaminated by noises and motion artifacts, which will influence the estimation performance of gait phase. In addition to muscles’ electrical potential, there exist muscle deformations during limb motion. The capacitive sensors have been designed and used in prosthesis and exoskeleton (Zheng et al., 2014). In our previous study, we conducted the preliminary study of the continuous gait phase estimation based on capacitive sensing signals with the AOs (Xu et al., 2020a). The previous study just conducted the continuous gait phase estimation on one amputee at normal walking speed (Xu et al., 2020a). In this study, we improved the capacitive sensing design and extended the study on two amputees at different walking speeds and on different ramps to realize the offline adaptive estimation of continuous gait phase. This paper is organized as follows. First, we introduce the related studies and research progress in “Introduction”. Then, we introduce the robotic transtibial prosthesis, capacitive sensing design, experimental protocol, signal processing, and evaluation method in section “Materials and Methods”. Then, the result, discussion, and conclusion are presented.

## Material and Method

### Robotic Transtibial Prosthesis

**Figure 1.**
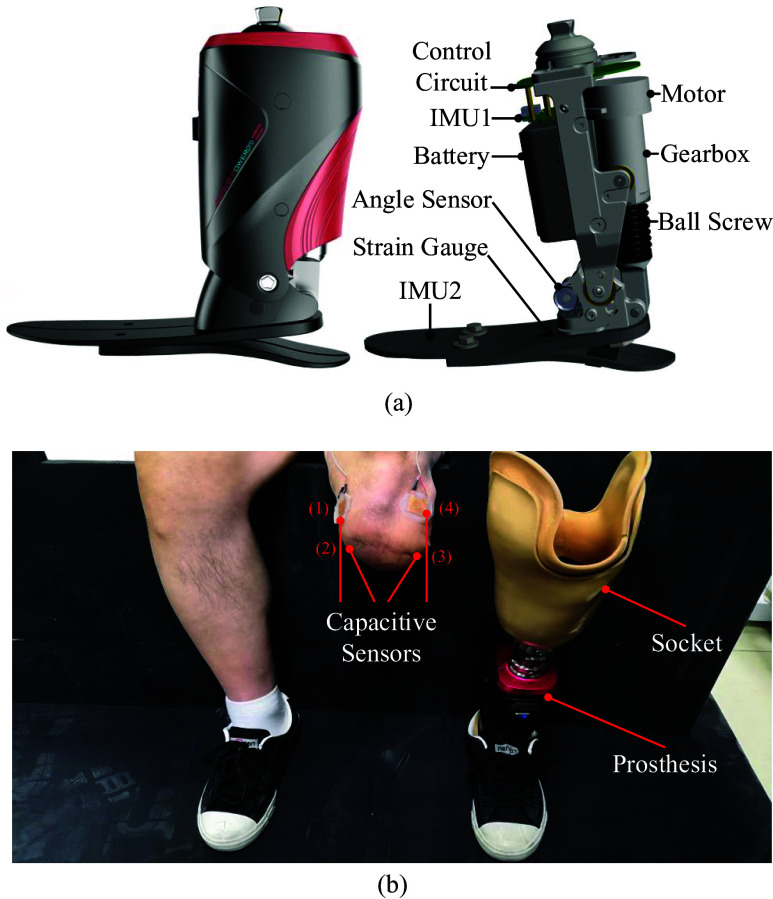
The designed robotic transtibial prosthesis and wearing diagram. (a) The designed robotic transtibial prosthesis includes the mechanical, sensing, and actuation components. (b) The wearing diagram. Four capacitive sensors (labeled by 1, 2, 3, and 4) are placed on the residual limb.

A robotic transtibial prosthesis (developed by Peking University, Beijing, China) was used in this study, and its details are shown in Figure 1a. The sensing units of prosthesis were comprised of one angle sensor, one strain gauge, and two IMUs. One angle sensor was positioned on the prosthetic ankle to detect ankle’s rotation. The strain gauge was positioned on the prosthetic foot to detect the interaction between the prosthesis and ground during walking, and the gait phase could be detected based on the strain signals. In this study, we adopted the position control (to return to the equilibrium position) in the swing phase and damping control (to provide assistance) in the stance phase, respectively (Wang et al., 2015; Feng and Wang, 2019). Two IMUs were mounted on the prosthetic shank and foot to measure acceleration, angle, and angular velocity information. More details of this robotic transtibial prosthesis could be seen in some previous studies (Wang et al., 2015; Feng and Wang, 2019). Capacitive sensing method could measure muscles’ deformations, and its principle has been described in a previous study (Zheng et al., 2014). Four capacitive sensors were placed on the residual lower limb (labeled as 1, 2, 3, and 4 in Figure 1b). There was a liner placed over the residual lower limb and capacitive sensors, after which the socket was fitted. The capacitive sensors could record the deformation which was caused by contraction and relaxation of residual muscle and the interaction of the residual limb, liner, and the socket.

### Capacitive Sensor Design

#### Capacitive signal measurement

In this study, capacitive sensing method is used to measure the lower-limb deformations during human walking. The calculation formula of capacitive signal is as follows:
(1)

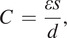

where the 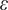 is the dielectric constant of the dielectric layer, and the 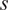 and 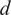 are the relative area of the two electrodes and the distance between the two electrodes, respectively. The capacitor is attached to the surface of the limbs. When people walk, the muscles of the limbs will be in contraction and relaxation. For the prothesis wearer, there is the interaction among the residual limb, liner, and the socket. Both of these will lead to the changes in the distance between the two electrodes or/and the relative area of the two electrodes, and finally, cause the changes in the capacitive signal.

**Figure 2.**
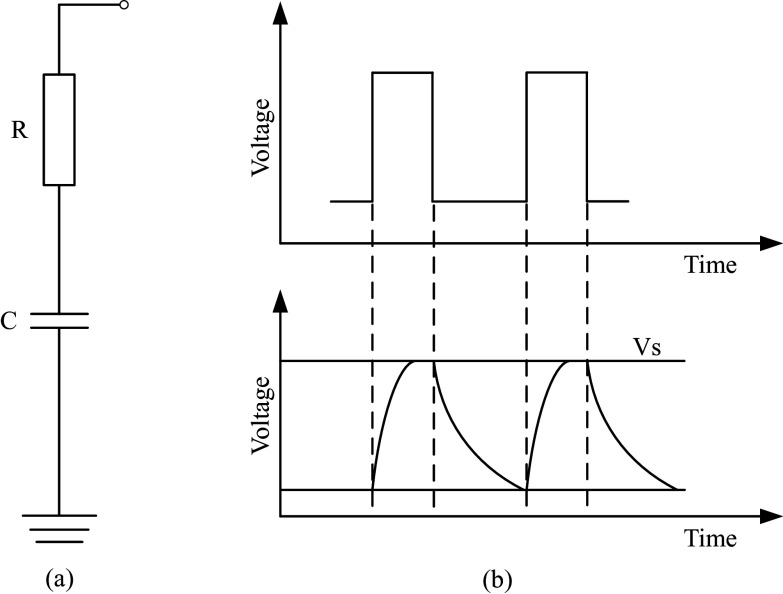
The capacitive signal measurement system. (a) The measurement circuit of capacitive signal and (b) The measurement principle of capacitive signal.

Capacitive sensing system is used to record the change of capacitance signal, mainly including the front end (the capacitor) and the back end (the signal processing circuit). In the measurement of capacitance signal, the basic principle is to record the charging and discharging time and then calculate the capacitance value, as shown in Figure 2. In the measurement circuit, the capacitor 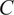 is in series with the resistor 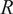 . When the square wave signal is applied in the circuit, it will charge the capacitor at the rising edge to the highest voltage 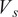 . At the falling edge, the capacitor starts to discharge, and the voltage of the capacitor can be formulated as follows:
(2)

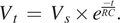

When the capacitor voltage 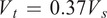 , the corresponding discharge time 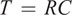 . The discharge time is proportional to the capacitance value, so the corresponding capacitance value can be obtained by recording the discharge time. The measurement circuit also contains a reference capacitor (its value is 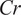 ). When measuring capacitive signal, the ratio of the capacitance value to be measured to the reference capacitance value can be calculated according to the time, and then the capacitance value to be measured can be calculated. The charge–discharge time ratio is recorded by time-to-digital converter (TDC) module with an accuracy of 21 bit and a range of 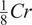 –8 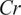 . The capacitance value of the reference capacitor is 100 pF in this study, and the measurement range of this capacitor signal measurement circuit is 12.5 pF–800 pF. The signal sampling frequency is 100 Hz.

#### Capacitive sensor fabrication

In this study, we used the copper electrodes to form capacitors with Ecoflex (Aliphatic Aromatic Copolyesters). This capacitor could withstand stretching, bending, and other deformations. This capacitor was composed of two copper electrodes and the dielectric layer (Ecoflex) between the two electrodes. Besides, the entire capacitor was wrapped in Ecoflex. When people wore this capacitive sensor to measure the capacitive signals, the copper electrodes of the capacitor were not in directly contact with the skin. Thus, the noncontact measurement of muscle deformation could be realized.

**Figure 3.**
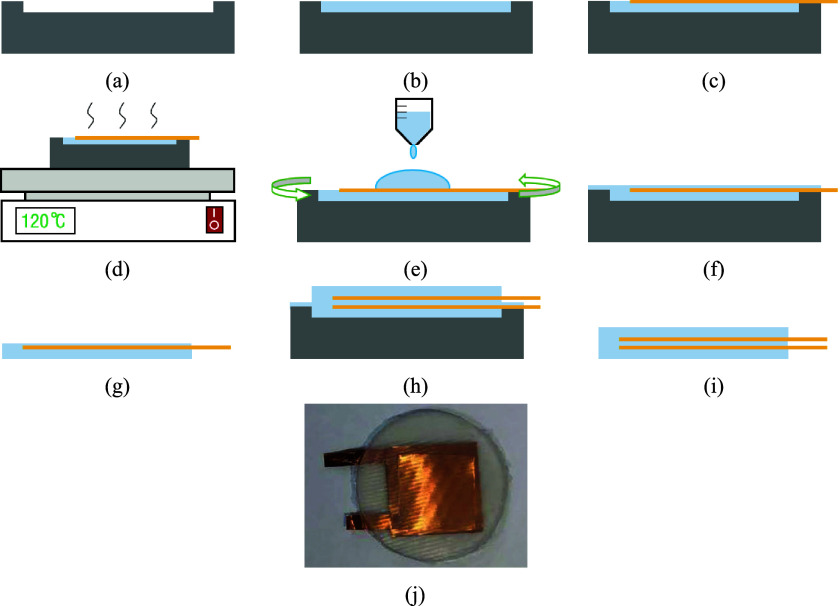
The fabrication process of capacitor. (a) printing 3D molds, (b) pouring Ecoflex, (c) placing copper electrodes, (d) curing, (e) spin-coating Ecoflex, (f) curing, (g) removing the mold, (h) bonding the two-halves structures, (i) curing and removing the mold, and (j) capacitor picture.

The fabrication process of capacitor are shown in Figure 3. First, one mold was made by 3D printing, as shown in Figure 3a. Then, the Ecoflex was poured into the mold, as shown in Figure 3b. When the EcoFlex was not cured, one copper electrode (thickness: 50 μm) was put on the Ecoflex, as shown in Figure 3c. Next, the EcoFlex and electrode needed to be cured at 120–140°C for about 5 min, as shown in Figure 3d. Then, we coated the cured EcoFlex and electrode with a new Ecoflex layer (thickness: 50 μm), as shown in Figure 3e. The next step was curing and demolding. After this, half of the capacitor was finished, as shown in Figure 3f,g. We could get the two-halves of the capacitor by repeating the fabrication process (from Figure 3a to e). By bonding the two-halves structures (as shown in Figure 3h and then curing and removing the mold (as shown in Figure 3i), we could get an entire capacitor. The finished capacitor is shown in Figure 3j. The radius and thickness of fabricated capacitor are approximately 1 cm and 2 mm, respectively. Each copper electrode is about 1.2 to 1.5 cm in length, 1.2 to 1.5 cm in width, and about 0.05 mm in thickness.

### Experimental Protocol

Two transtibial amputees joined in this study as subjects and their detailed information are listed in Table 1. Each subject wore his customized prosthetic socket and liner, and the socket was connected with the robotic transtibial prosthesis by an adapter. Both the two subjects have provided written informed consent, and the experiments have been approved by the Local Ethics Committee of Peking University.

**Table 1.** The information of two transtibial amputation subjects

	Gender	Age	Weight (kg)	Height (cm)	Years post-amputation	The amputation side	Residual limb length ratio (%)
Subject 1	Male	31	72	171	10	Right	33
Subject 2	Male	54	70	170	18	Left	40

The research tasks were comprised of two experiments. The first experiment was to conduct the offline estimation of continuous gait phase at each subject’ different walking speeds (speed experiment). In the speed experiment, subjects walked on the treadmill at their self-selected three speeds (slow, normal, and fast). The second experiment was to conduct the offline estimation of continuous gait phase on different ramps (ramp experiment). In the ramp experiment, subjects walked on the treadmill, and the treadmill inclination varied over five different angles (10°, 5°, 0°, −5° and −10°). Subjects walked on the inclined treadmill at their normal speed. The offline estimation of continuous gait phase was conducted based on the collected capacitive signals and strain signals of prosthetic foot during subjects’ walking at different speeds and on different ramps.

### Offline Estimation of Continuous Gait Phase

**Figure 4.**
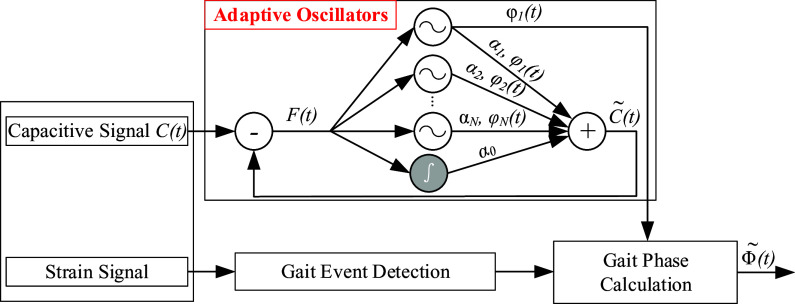
The framework of gait phase estimation (adapted from Gams et al., 2009; Yan et al., 2017; Xu et al., 2020b). The capacitive sensing signals are input to the AOs. The strain signals of prosthetic foot are used to detect gait events.

The framework of offline estimation of continuous gait phase is shown in Figure 4, which consist of (1) the AOs and (2) the gait event detection. The AOs have been widely used in cyclical movements (Ronsse et al., 2011). Some studies have conducted the continuous gait phase estimation based on the AOs (Yan et al., 2017; Zheng et al., 2017). In this study, the inputs of the AOs were the capacitive sensing signals of residual limb. The collected raw capacitive sensing signals contained drifts and noises: low-frequency drifts (lower than 0.1 Hz), random impulses, and high-frequency noises (Zheng et al., 2014). In this study, we designed three filters in series (a median filter, a first-order DC-notch filter, and a second-order 10-Hz low-pass Butterworth filter) to regulate the raw capacitive sensing signals by removing drifts and noises. The gait event detection was conducted based on the strain signals of prosthetic foot by the threshold rule. In this study, the detected heel strike was the start point of each gait cycle and it was also corresponding to 0 rad gait phase.

In Figure 4, the inputs of the AOs are capacitive sensing signals ( 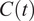 ), which could be decomposed into different harmonics. The outputs are the estimated signals ( 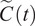 ). The error 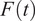 ( 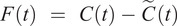 ) between the actual signal (input) and the estimated signal (output) drove the evolution of the oscillator (Yan et al., 2017). The 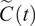 could be denoted as follow:
(3)

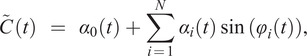

where the 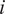 denoted the 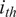 harmonic ( 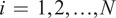 and 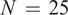 in this study). The other variables and their principle formulations were as follows:
(4)

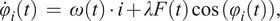



(5)

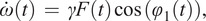



(6)

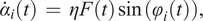



(7)

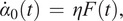

where 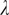 , 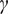 and 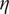 were the learning rates corresponding to phase ( 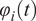 ), frequency ( 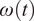 ), and amplitude ( 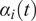 ), respectively.

The lower-limb locomotion could be viewed as periodic or quasi-periodic. Therefore, we defined the continuous gait phase corresponding to the interval [0, 2 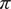 ) rad. The acquired phase 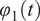 (in Figure 4) based on the AOs was normalized into the interval [0, 2 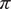 ), and the normalized phase was denoted as 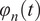 (in Figure 4):
(8)

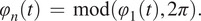

In this study, we defined the heel strike as the start point (0 rad) of one gait cycle. The 0-rad phase should be matched with each heel strike at timing 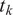 in gait cycles, so there might exist phase error 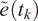 between the estimated phase 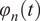 at 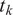 and 0 rad. The final estimated gait phase 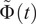 could be denoted as follow:
(9)

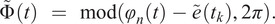

More details about the preprocess of 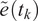 could be seen in the study by Yan et al., 2017). As was known that the gait phase increases forward within each gait cycle, we could revise the current estimated gait phase by comparing it with the last estimated gait phase(s) to make sure the monotonic increasing feature of gait phase forward in each gait cycle.

### Performance Evaluation

The variance ratio ( 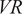 ) could be used to analyze the repeatability of gait signals waveforms over gait cycles (Erni and Colombo, 1998; Hwang et al., 2003; Godiyal et al., 2018). 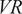 was formulated as follows (Hershler and Milner, 1978):
(10)

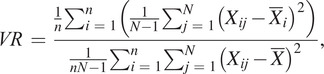

where 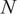 denoted the number of gait cycles in this study. For each gait cycle, signals were normalized by interpolation and had a fixed length ( 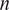 , 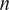 was 1000 in this study). The 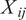 was the 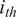 capacitive signal value in 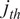 gait cycle. The 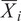 was the mean of signals at 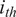 data point over 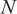 gait cycles, and the 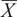 was the mean of 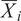 over the gait cycle. The 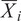 and 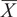 were formulated as
(11)

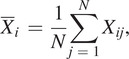



(12)

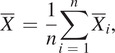

The 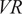 could measure the degree of dispersion of data. The smaller the 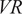 was, the better the signal repeatability was. If the 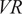 < 0.3, it represented a good signal repeatability (Nilsson and Thorstensson, 1987).

To evaluate the estimation performance, the root-mean-square error 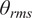 between the estimated phase and the ground truth was used, which could be formulated as follows:
(13)

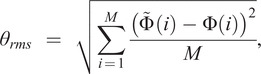

where 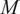 denoted the sample number in one gait cycle, the 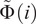 denoted the estimated 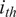 gait phase and the 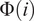 was the actual 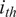 gait phase in one gait cycle. Besides, we also introduced the ratio ( 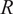 ) of the root-mean-square error ( 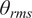 ) in one gait cycle. The 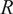 could be calculated as follows:
(14)

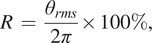

where 2 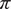 was the gait phase length of one gait cycle.

## Experimental Results

### Capacitive Signal Repeatability

**Figure 5.**
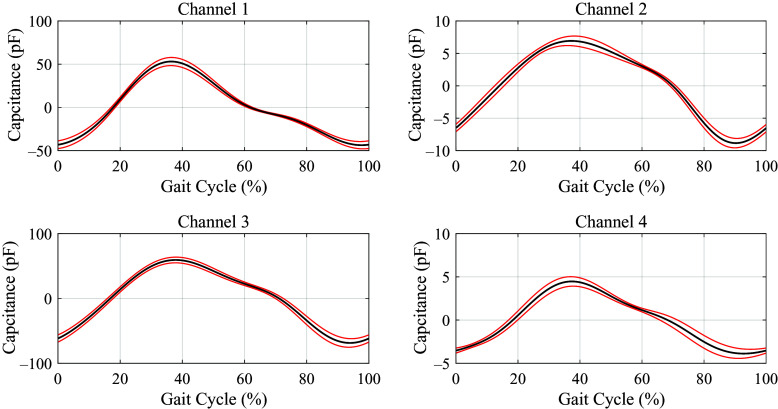
The normalized capacitive sensing signals. (a)–(d) represent 4 channels’ capacitive sensing signals. The black solid curves denote the mean of capacitive sensing signals of 50 gait cycles. The red solid lines denote the mean ± standard deviation. The gait cycle starts at the heel strike and ends at the next heel strike, corresponding to the gait percent from 0 to 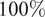 (shown in the horizontal axis). Data come from subject 1 walking at normal speed.

The capacitive sensing system recorded the muscles’ deformation of residual limbs during walking. We conducted capacitive signal normalization process to evaluate signal’s repeatability. Taking the level ground walking at normal speed as an example, the four channels’ filtered capacitive sensing signals were normalized into one gait cycle, as shown in Figure 5(a)–(d). The black solid curves denoted the mean and the two red solid curves denoted mean + standard deviation and mean – standard deviation of capacitive sensing signals. The areas between the two red solid curves reflected the signals’ differences.

We used 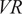 to further evaluate the repeatability, and the results are shown in Tables 2 and 3. For subject 1, the maximum and minimum of 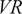 were 0.003 and 0.151 (in Table 2), respectively. For subject 2, the maximum and minimum of 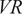 were 0.005 and 0.090 (in Table 3), respectively.

**Table 2.** The 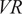 of four channels’ capacitive signals (subject 1)

		C1	C2	C3	C4
Speed	Slow	0.043	0.007	0.042	0.006
	Normal	0.035	0.007	0.027	0.005
	Fast	0.043	0.007	0.034	0.004
Ramp	10°	0.034	0.025	0.151	0.007
	5°	0.047	0.005	0.039	0.003
	0°	0.035	0.007	0.027	0.005
	−5°	0.021	0.006	0.022	0.004
	−10°	0.080	0.021	0.023	0.008

*Note.* C1, C2, C3, and C4 denote the 4 channels’ capacitive sensing signals.

**Table 3.** The 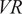 of four channels’ capacitive signals (subject 2)

		C1	C2	C3	C4
Speed	Slow	0.013	0.014	0.044	0.075
	Normal	0.013	0.014	0.044	0.075
	Fast	0.008	0.008	0.010	0.044
Ramp	10°	0.014	0.013	0.018	0.080
	5°	0.090	0.006	0.012	0.034
	0°	0.011	0.014	0.013	0.030
	−5°	0.005	0.005	0.008	0.019
	−10°	0.007	0.010	0.015	0.050

*Note.* C1, C2, C3, and C4 denote the 4 channels’ capacitive sensing signals.

### Gait Event Detection

**Figure 6.**
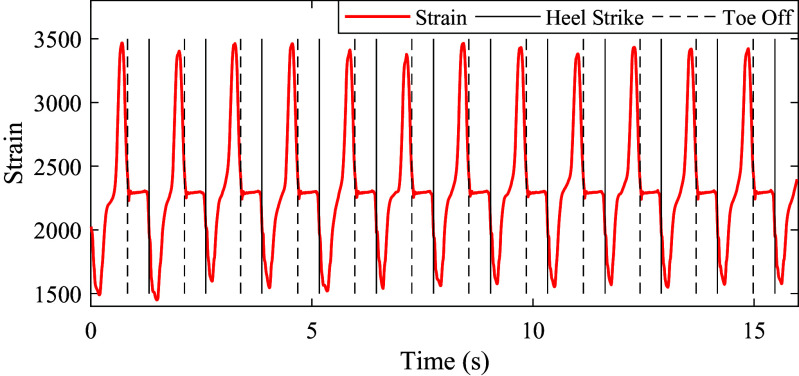
The strain signals of prosthetic foot at normal walking speed (subject 1). The red solid curve denotes the strain signal. The black dashed and solid lines denote the gait events: heel strike and toe off, respectively.

The strain signals of prosthetic foot during level-ground walking at normal speed is shown in Figure 6. The strain signals had different characteristics in different gait phases. In the stance phase, there were interactions between prosthetic foot and the ground. Therefore, the strain signals showed obvious changes. In the swing phase, there were no interactions between prosthetic foot and the ground. Therefore, the strain signals showed few changes. By analyzing the characteristics of strain signals, we could realize the gait events (heel strike and toe off) detection.

### Offline Estimation of Continuous Gait Phase

**Figure 7.**
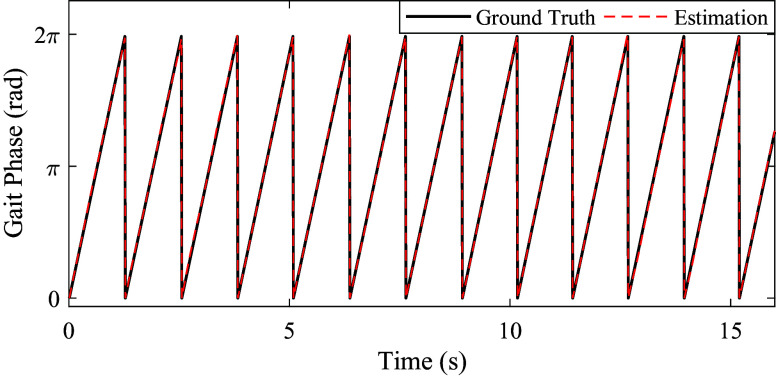
The estimated continuous gait phase and the ground truth. The black solid denotes the estimated gait phase, and the red dashed denotes the ground truth.

The AOs have three learning rate parameters 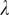 , 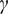 , and 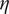 (as shown in equations (4), (5), and (6)), corresponding to the phase ( 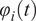 ), frequency ( 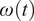 ), and amplitude ( 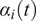 ), respectively. In this study, they were set as 0.15, 0.50, and 0.60, respectively. The values of the three parameters were kept constant for different subjects waking at different walking speeds and on different ramps. Based on the capacitive sensing signals and the strain signals, the continuous gait phase estimation result is shown in Figure 7.

**Figure 8.**
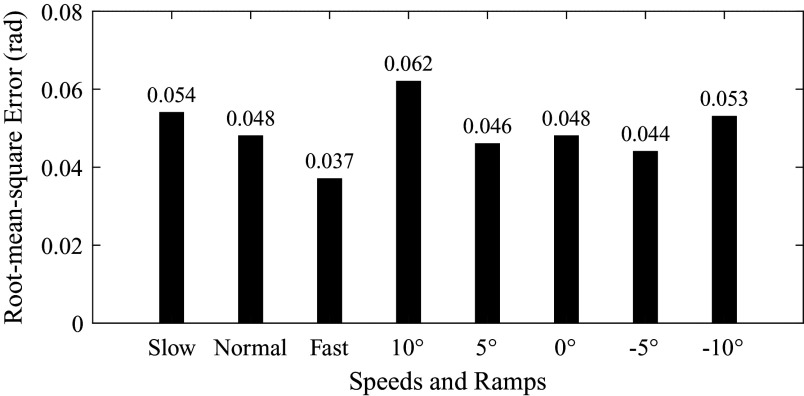
The root-mean-square error between the estimation result and the ground truth (subject 1). The numbers on the top of bars represent the error values.

**Figure 9.**
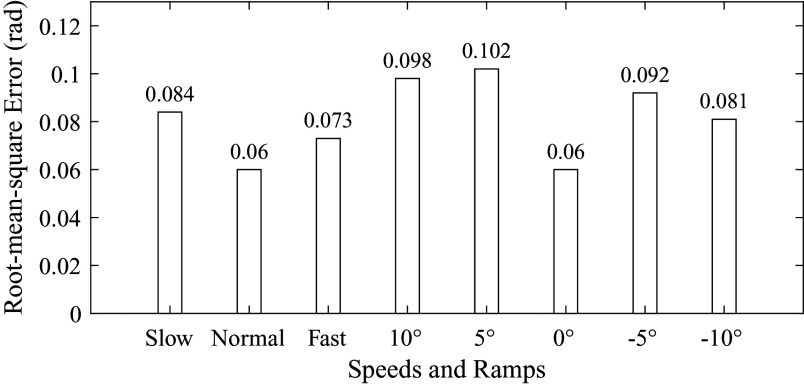
The root-mean-square error between the estimation result and the ground truth (subject 2). The numbers on the top of bars represent the error values.

The root-mean-square error ( 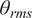 ) between the estimation result and the ground truth is shown in Figures 8 and 9. For subject 1, the root-mean-square errors corresponding to different speeds and ramps were 0.054 rad, 0.048 rad, 0.037 rad, 0.062 rad, 0.046 rad, 0.048 rad, 0.044 rad and 0.053 rad, respectively. The maximum was 0.062 rad (corresponding to 10° ramp), and the minimum was 0.037 rad (corresponding to fast speed). For subject 2, the root-mean-square errors were 0.084 rad, 0.060 rad, 0.073 rad, 0.098 rad, 0.102 rad, 0.060 rad, 0.092 rad and 0.081 rad. The maximum was 0.102 rad (corresponding to 5° ramp), and the minimum was 0.060 rad (corresponding to normal speed and 0° ramp).

The average and standard deviation of root-mean-square errors and ratios are listed in Table 4. The errors were 0.069 ± 0.021 rad, 0.054 ± 0.008 rad, 0.055 ± 0.025 rad, 0.080 ± 0.025 rad, 0.074 ± 0.039 rad, 0.054 ± 0.008 rad, 0.068 ± 0.034 rad and 0.067 ± 0.019 rad, corresponding to walking at different speeds and on different ramps, as shown in Table 4. The maximum error and its corresponding ratio were 0.080 rad and 1.27% (10° ramp). The minimum error and its corresponding ratio were 0.054 rad and 0.86% (normal speed and 0° ramp).

**Table 4.** The root-mean-square error ( 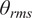 ) and ratio ( 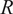 ) (mean ± Std) of continuous gait phase estimation

		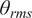 (rad)	*R* (%)
Speed	Slow	0.069 ± 0.021	1.09 ± 0.34
	Normal	0.054 ± 0.008	0.86 ± 0.13
	Fast	0.055 ± 0.025	0.88 ± 0.40
Ramp	10°	0.080 ± 0.025	1.27 ± 0.40
	5°	0.074 ± 0.039	1.18 ± 0.63
	0°	0.054 ± 0.008	0.86 ± 0.13
	−5°	0.068 ± 0.034	1.09 ± 0.54
	−10°	0.067 ± 0.019	1.07 ± 0.31

## Discussion

The finite state machine control method is to set different control strategies and parameters in different gait states. This relies on the accurate detection of several discrete gait events (heel strike, toe off, and so on) to trigger the transition of control strategy from one gait state to another gait state, which can decrease the smoothness and robustness of robot’s locomotion (Villarreal et al., 2017; Yan et al., 2017). One alternative is control based on the estimation of continuous gait phase. This control method is not limited to the states’ transitions to change control strategy in each gait cycle, which is helpful to improve the smoothness and robustness of robot’s motion and then enhance the assist functions in people’s activities. Therefore, it is important to realize an accurate estimation of continuous gait phase to control lower-limb wearable robot. Hereby, this study has focused on the offline adaptive estimation of continuous gait phase of robotic transtibial prosthesis.

The main contribution of this study is that we propose a capacitive sensing method to record the muscle’s deformation to realize the adaptive estimation of continuous gait phase during different steady-state motions. To achieve this goal, we have designed the capacitive sensors and the framework of continuous gait phase estimation based on gait event detection and AOs.

### Capacitive Sensing Signals

There may exist stride-to-stride variation for lower-limb amputees, even in the steady walking mode, which can cause sensing signal variation. To estimate the gait phase, the input sensing signal is required to be quasi-periodic with the gait cycles. Noncontact capacitive sensing method has quite small signal variation, good signal repeatability, and periodicity in lower-limb locomotion, which has been validated in a previous study (Zheng et al., 2014). The capacitive sensors are attached to the residual limb, and there is a liner placed over them, after which the socket is fitted. The capacitive sensors can record the deformation which is caused by the contraction and relaxation of residual muscle and the interaction of the residual limb, liner, and the socket. For the two subjects, the maximum of 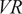 is smaller than 0.3, which means the capacitive sensing signals have good repeatability and periodicity. This provides benefits for the estimation of continuous gait phase. The strain signals of prosthetic have different characteristics in different gait phases. By analyzing the characteristics of strain signal, we could realize the accurate gait events (heel strike and toe off) detection. The detected heel strike is the start of one gait cycle and corresponds to the reset 0 rad.

### Offline Estimation of Continuous Gait Phase

We have conducted the offline estimation of continuous gait phase based on the AOs. The initial values of parameters in AOs are as follows. The initial values of 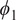 and 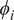 ( 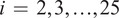 ) are 0.5 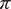 and 1, respectively. The initial value of 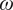 is 0.8*2 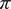 (i.e., 1.6 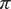 ). The initial values of 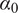 and 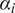 ( 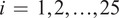 ) are 0 and 1, respectively. The AOs adapt over time based on these parameters. The AOs have three learning rate parameters 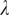 , 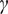 _,_ and 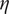 (as shown in equations (4), (5), and (6)) that are kept constant for different subjects waking at different walking speeds and on different ramps. This eases parameter tuning difficulty and reduces the dependence on the experimenters. In this study, our analysis is limited to once the AOs have converged. Actually, the AOs do not perform good linear characteristics and the estimation errors are big prior to convergence and become small over time. The convergence of AOs takes about three to five gait cycles with these learning parameters. In this study, each trial takes about 3 min (walking lasts about 3 min), and the amount of each trial is about 100 gait cycles used in the analysis. The maximum and minimum errors (and ratios) of offline estimation of continuous gait phase are 0.080 rad (1.27%) and 0.054 rad (0.86%) corresponding to slow and fast speeds, respectively. The results of this study are comparable with the study by Zheng et al. (2017), which has conducted the estimation of continuous gait phase based on the AOs on healthy people. Our previous study has conducted the estimation based on the AOs using inertial sensing signals (Xu et al., 2020b). Compared with this study, there is no significant difference ( 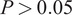 ) in estimation performance based on the capacitive sensing signals and inertial sensing signals.

Thatte et al. (2019) have conducted the continuous gait phase estimation based on the extended Kalman filter method, and the root-mean-square phase error (ratio) is more than 2.0%. Our study based on AOs can achieve less error, and the biggest error ratio is 1.27% ± 0.40%. Seo et al. (2019) have also conducted a similar study. Though their study has not given specific numerical results, their continuous gait phase estimation curve shows not good linear characteristics (Seo et al., 2019). Our study has illustrated good linear characteristics of the estimated continuous gait phase, as shown in Figure 7. Compared with the extended Kalman filter-based and RNN-based methods (Thatte et al., 2019; Seo et al., 2019), this study has achieved better estimation performances.

### Impacts of Estimation Errors

The lower-limb locomotion can be viewed as periodic or quasi-periodic, but it is not absolutely periodic. Though the capacitive sensing signals have good repeatability and periodicity ( 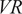 < 0.3), there still exist signals’ differences among different gait cycles. The signals’ differences will cause errors between the estimated gait phase and the ground truth, and these errors will cause some different impacts on prosthesis control (Villarreal et al., 2017; Rezazadeh et al., 2019; Embry and Gregg, 2021). If some errors are very small, we can think they may cause small impacts on prosthesis wearers’ walking. Sometimes, some errors may cause few impacts. For example, some errors occurring at the prosthetic swing stage may not decline the prosthetic assist functions, since the goal of prosthesis control during the swing stage is to reset prosthesis for the next heel strike and the prosthesis has no interaction with the ground in the swing stage. On the other hand, some errors may cause big impacts on prosthesis wearers’ walking, for example, these errors that are big or occur at some gait stage (e.g., the whole stance stage). We need to pay more attention to these errors. In short, the impacts caused by errors vary. Therefore, errors need to be carefully dealt with by taking multifactors (such as the error value (big or small), the stage that error occurs at, the designed control strategies, and so on) into consideration when continuous gait phase estimation is used in prosthesis control.

### Limitations and Future work

The continuous gait phase estimation still needs to be further developed. More amputees need to be recruited to analyze the effectiveness and adaptability of the continuous gait phase estimation. Besides, it is practical to conduct the study on the nonsteady-state motion in future work. There are few studies on nonsteady-state motion up to now. The inherent synchronization properties of the AOs have provided advantages in continuous gait phase estimation of steady-state motion, and it has shown some adaptations to different steady-state motions. Whether the AOs work and how it performs on nonsteady-state motion still need to be further studied. In addition, it is also a way to adopt another method or design a new algorithm to realize the estimation of nonsteady-state motion.

The current continuous gait phase estimation is offline in this study. We need to embed the continuous gait phase estimation algorithm into the prosthetic control circuit to realize the online estimation, and then combine the online estimation with prosthesis control to improve the assist performance. The computation cost needs to be considered to meet the online requirement in the next work. We should choose a strong computing hardware to embed the online algorithm to execute online computation, and the computation must be fast and the computing time should less than the sample interval, that is, the computation must be finished before the arrival of a new sample. In our study, the sample frequency is 100 Hz and the sampling interval are 10 ms. This means the online computation must be finished within 10 ms. If so, the delay time caused by computing will cause little influence since it accounts for less than 1.0% of one stride time (the average stride time is about 1.2 to 1.3 s in this study). Besides, the mapping relationships between the joint torque (joint angle, angular velocity, etc.) and the continuous gait phase need to be well-built. According to the online estimation result, a future control algorithm will instruct the robotic prosthesis to make a response (for example, driving the motor to output specific joint torque continuously). In addition, errors need to be carefully dealt with in future work, and the impacts caused by errors vary and need to be further studied when continuous gait phase estimation is used in prosthesis control.

## Conclusion

In this study, we have conducted the offline adaptive estimation of continuous gait phase in robotic transtibial prosthesis based on the residual muscles’ capacitive signals. The offline estimation experiments of continuous gait phase are performed at different speeds (slow, normal, and fast) and on different ramps (10°, 5°, 0°, −5°, and −10°). Based on the strain signals of the prosthetic foot and the capacitive signals of the residual limb, the maximum and minimum estimation errors are 0.80 rad and 0.054 rad (the corresponding ratios in 1 gait cycle are 1.27 and 0.86%), respectively. The results indicate that our proposed method is an effective method to estimate the continuous gait phase and it has adapted to different walking speeds, ramps, and subjects. The estimation of continuous gait phase provides a basis for the continuous control of robotic transtibial prosthesis.

## Data Availability

All data and media materials that are available are mentioned within the article’s main text with links to their online repositories in footnotes.
